# Pain Management During Intrauterine Device Insertion in Nulliparous Women: A Scoping Review

**DOI:** 10.7759/cureus.71774

**Published:** 2024-10-18

**Authors:** Kami Mukenschnabl, Emily A Ina, Toni Bacoat-Jones

**Affiliations:** 1 Obstetrics and Gynecology, Nova Southeastern University Dr. Kiran C. Patel College of Osteopathic Medicine, Davie, USA; 2 Osteopathic Medical School, Nova Southeastern University Dr. Kiran C. Patel College of Osteopathic Medicine, Fort Lauderdale, USA; 3 Faculty of Foundational Sciences, Nova Southeastern University Dr. Kiran C. Patel College of Osteopathic Medicine, Clearwater, USA

**Keywords:** analgesic, cervical block, intrauterine device insertion, iud insertion pain management, nulliparous female

## Abstract

This study investigates the various methods of pain management during the insertion of intrauterine devices (IUDs) in nulliparous women. Currently, the only recommended method of pain management is 800 mg of ibuprofen taken one hour before insertion of the IUD. However, women continue to experience pain during the procedure. A scoping review was conducted using CINAHL, Medline, Web of Science, and Embase with inclusion criteria being English peer-reviewed articles from the last 10 years, involving nulliparous women of at least 18 years of age. The research reveals that management to minimize pain during IUD insertion can include oral analgesics, cervical blocks, and cervical softening and dilation with prostaglandins. The effect of pain management when using these techniques was further examined throughout the various steps of IUD placement, including cervical grasping, IUD insertion, and post IUD insertion. Ibuprofen is the current recommended analgesic; however, studies show that there was no significant reduction in pain found when ibuprofen is used. Alternatively, 500 mg of naproxen sodium taken prior to IUD insertion showed a significant reduction in post-IUD insertion pain (p=0.01) but did not show any significant reduction in pain during cervical grasping or during IUD insertion into the uterus. Cervical blocks using 1% lidocaine were shown to decrease pain during cervical gripping (p=0.002) and IUD insertion compared to the control group (p=0.005). The results of cervical blocks differed based on whether 1% lidocaine was injected or if a 2% lidocaine gel was used, but no significance was shown. Furthermore, cervical softening and dilation with dinoprostone 3 mg and misoprostol 3 mg demonstrated a reduction in pain during all stages of IUD insertion and after insertion (p<0.01). Pharmacological interventions with oral analgesics, lidocaine, and prostaglandins, such as dinoprostone and misoprostol, have all demonstrated some level of pain control during the IUD insertion procedure, but the use of prostaglandins and 2% lidocaine gel has been demonstrated to have the most clinically significant effect on pain control. Additionally, there has been some research examining the impact of verbal analgesics, which involves the provider using a calm, soothing voice and slow speech to put the patient at ease, and the role that anxiety about IUD insertion can influence pain, but further research is needed to determine its significance. This research provides valuable insight into enhancing the improvement of pain during and after the insertion of IUDs for nulliparous women.

## Introduction and background

Intrauterine devices (IUDs) are not only one of the most effective contraceptives, with a 99.9% success rate, but they are also one of the most user-friendly options for birth control as they do not need to be replaced for five to eight years after they are inserted. Current data show that 10.4% of women aged 15-49 years old in the United States use an IUD as a contraceptive [1]. However, despite this, more than 70% of women who are nulliparous, and receiving their first IUD, experience moderate to severe discomfort during the process of IUD insertion [2]. Current guidelines for IUD insertion recommend the use of 800 mg of ibuprofen one hour prior to IUD insertion. Interestingly, studies also illustrated that 800 mg of ibuprofen does not provide effective pain relief to patients during or after an IUD insertion procedure [2]. Guidelines also state there is an option to use a paracervical block, yet there is no absolute indication for the use of lidocaine via a paracervical block due to the contradictory results of this method in various studies [3]. More recently, there have been some institutions utilizing general anesthesia to alleviate the pain associated with the IUD insertion process. Studies have found that, although general anesthesia could reduce the pain during the IUD insertion, pain and cramping may still be present post IUD insertion [3]. Therefore, further research on methods of pain control for the IUD insertion process needs to be studied to improve patient care from the provider's standpoint and improve patient satisfaction with long-acting contraceptives, such as IUDs. The aim of this systematic review is to investigate the various methods of pain control used during IUD insertion to determine the most effective approach to minimize pain during the procedure in nulliparous females.

## Review

Material and methods

The scoping review methodology was performed in accordance with the Joanna Briggs Institute (JBI) standards, ensuring a thorough examination of the literature regarding IUD insertion, pain management, and nulliparous women. The JBI methodology utilizes an evidence-based approach that considers the feasibility, appropriateness, meaningfulness, and effectiveness (FAME) of each article. The base search strategy was constructed by analyzing key terms in Covidence and from relevant articles in Cumulated Index in Nursing and Allied Health Literature (CINAHL), Medline, Web of Science, and Embase. The researchers then limited articles to those written between 2014 and 2024. To meet inclusion criteria, articles had to be peer-reviewed, written in English, and based on research studies taking place at any time within the last 10 years. Other criteria included women of at least 18 years of age, nulliparous, and receiving their first IUD with no contraindications, such as pelvic inflammatory disease and pregnancy.

Researchers performed the initial search on September 12, 2023, and used the Population, Intervention, Comparison, Outcomes, and Study (PICOS) design as a framework to formulate eligibility criteria. Initially, electronic databases such as CINAHL, Medline, Web of Science, and additional databases within Embase were searched for articles published within the last five years using the Boolean phrase (IUD insertion OR (iud AND insertion)) AND (pain control OR (pain AND control) OR analgesia OR anesthesia) AND (nulliparous OR nulliparity). However, there was an inadequate number of sources found. A second search expanded to the last 10 years, using the same Boolean phrase, of case reports, clinical trials, comparative studies, and randomized controlled trials, which yielded 240 articles. From the initial search, 139 duplicates were removed and 101 articles remained for screening. Additionally, researchers excluded 72 articles that did not meet inclusion criteria, such as multiparous women, or studies not conducted within the last 10 years, and two articles that could not be accessed. From the remaining 29 articles, the researchers were able to eliminate 13 resources due to the following reasons - two studies took place outside of the specified time frame, one study was not written in the English language, four studies had the wrong study design, and four studies included the wrong patient population, such as multiparous women. The reviewers then discussed each full-text article that was being considered for inclusion. After studies were screened and assessed in full text for eligibility, the researchers determined that 16 studies were able to be included in the review.

The two authors then conducted the third research stage independently using Covidence to organize the results of the 16 studies. The search strategy was reviewed with a physician to ensure that the search question was relevant to clinical obstetrics and phrased correctly. Data were extracted on article characteristics, types of intervention used during IUD insertion, and results of pain during various stages, including during and after IUD insertion. Researchers used Covidence to organize the summaries and analyses, including, population, study design, measures, and findings of the chosen articles.

**Figure 1:**
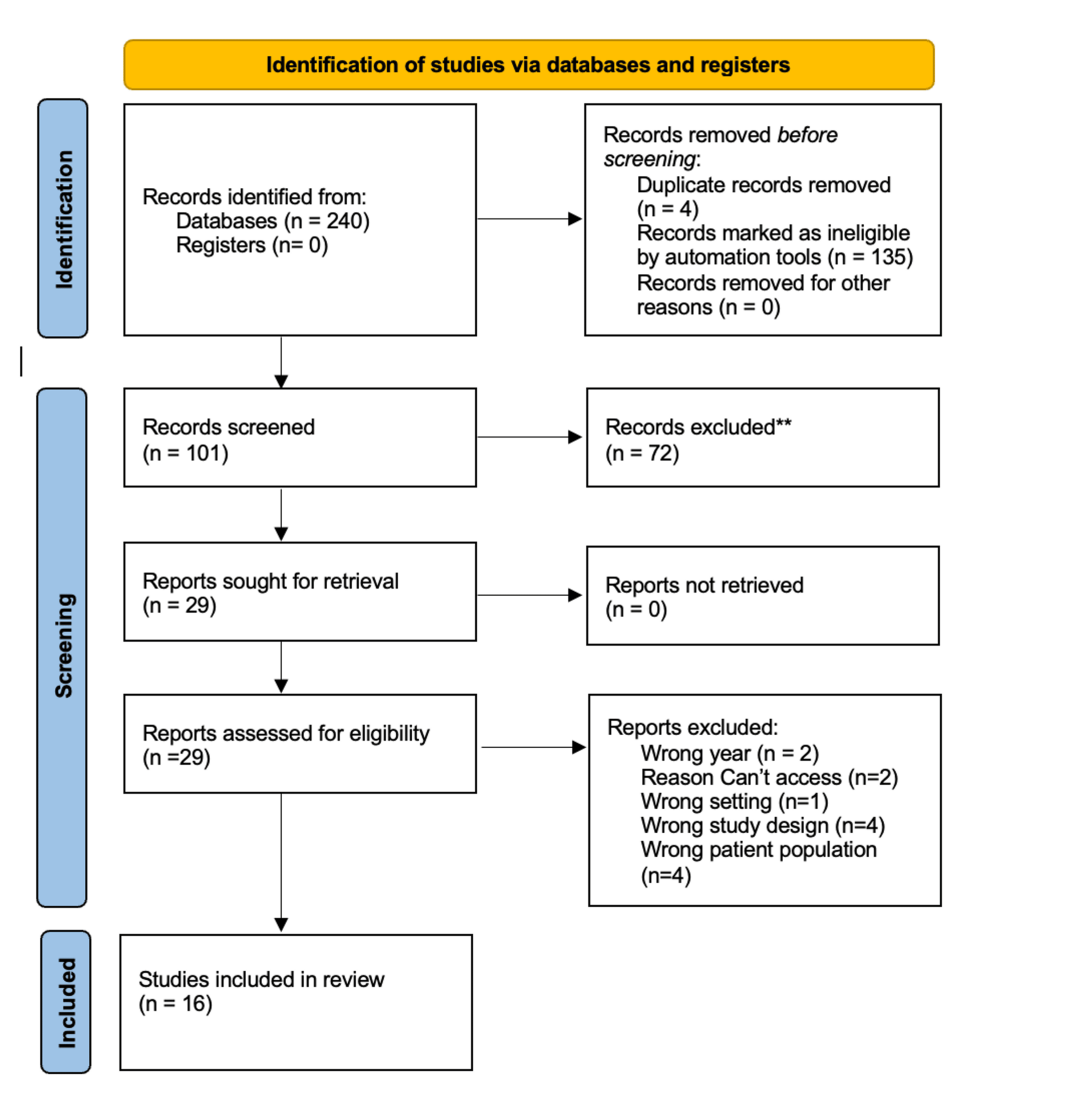
Preferred Reporting Items for Systematic Reviews and Meta-Analyses (PRISMA) Flow Diagram

Figure 1 shows the preferred reporting items for systematic reviews and meta-analyses (PRISMA) flow diagram to demonstrate the search methods utilized by the research team. Within the 16 studies of review, researchers grouped findings based on treatment modality, including oral analgesics, cervical blocks, and cervical softening with dilation.

Results

After further analysis of the results from the selected articles in this systematic review, researchers found that there were three main strategies used for the management of pain during IUD insertion. Most commonly, these included oral analgesics, such as ibuprofen or tramadol, cervical blocks with lidocaine, and softening and dilation of the cervix with the use of prostaglandins.

Characteristics of Included Studies

The 16 studies that were included in the study were conducted between 2014 and 2024. The selected studies were all randomized controlled trials (n=16). The included studies were completed in the United States of America (n=6), Egypt (n=4), Brazil (n=2), Israel (n=1), Switzerland (n=1), France (n=1), and Sweden (n=1). There were no notable demographic differences noted amongst any of the studies. The characteristics of the included studies are summarized in Table 1.

**Table 1:** Characteristics of Included Studies RCT: Randomized controlled trial; IUD: Intrauterine device

Authors	Title	Date of Publication	Type of Study	Country	Setting	Inclusion Criteria	Exclusion Criteria
Daykan et al. [4]	Verbal analgesia is as good as oral tramadol prior to intrauterine device (IUD) insertion, among nulliparous women: A randomized controlled trial	Mar 2021	RCT	Israel	Outpatient clinic	Nulliparous women ages 18-48 years, first-time IUD users	Previous pregnancy, current acute or recurrent pelvic inflammatory disease, acute cervicitis or acute vaginitis, current intraepithelial lesion, or any other genital malignancy, progesterone hypersensitivity, abnormal uterine bleeding, congenital or acquired uterine and anomaly, distorted uterine cavity (fibroid or polyp), impaired liver function and contraindication to tramadol.
Ngo et al. [5]	Naproxen sodium for pain control with intrauterine device insertion	Dec 2016	RCT	United States of America	Planned Parenthood League of Massachusetts health center	Women 18 years or older, premenopausal, presenting for insertion of any IUD type, and English-speaking or non-English-speaking. Limited to nulliparous women.	Women who were currently pregnant or had been pregnant within the previous 4 weeks, not eligible for IUD insertion per Planned Parenthood League of Massachusetts clinical protocols, were presenting to the clinic for IUD removal and reinsertion, had taken any pain medications within 12 hours of enrollment or long-acting narcotics within 48 hours of enrollment, had taken misoprostol within 24 hours or had any known allergy or contraindication to NSAIDs.
Mody et al. [6]	Paracervical block for intrauterine device placement among nulliparous women: A randomized controlled trial	Sep 2018	RCT	United States of America	Hospital	Nulliparous women 18 to 45 years old.	Pregnancy, any diagnoses of chronic pain issues, use of pain medication within 6 hours of enrollment, misoprostol administration within 24 hours of enrollment, history of prior IUD placement, or known contraindications to IUD placement.
Castro et al. [7]	Effect of intracervical anesthesia on pain associated with the insertion of the levonorgestrel-releasing intrauterine system in women without previous vaginal delivery: an RCT	Aug 2014	RCT	Brazil	Clinic Hospital	Nulliparous women 18-45 years old, with no previous IUD or vaginal delivery.	The exclusion criteria were women in categories 3 and/or 4 for LNG-IUS use according to the medical eligibility criteria of the World Health Organization (WHO) (WHO, 2009), illicit drug and/or alcohol users, women with allergies or contraindications to NSAIDs or lidocaine, acute or chronic pelvic pain of any etiology, abnormalities of the cervix (such as fibrosis or isthmus-cervical incompetence), previous abortion/miscarriage with or without uterine curettage, psychiatric disorders, and continued use of medications that could interfere with pain threshold.
Akers et al. [8]	Reducing pain during intrauterine device insertion: A randomized controlled trial in adolescents and young women	Oct 2017	RCT	United States of America	-	14-22-year-old nulliparous, not pregnant currently or in the prior 6 weeks, interested in Skyla IUD and English-speaking.	If they did not meet medical eligibility criteria for an IUD, contraindications to taking amino-amide anesthetics or non-steroidal anti-inflammatory agents, were unwilling to be randomized, were at high risk for pregnancy, used narcotics of benzodiazepines in the prior 24 hours, previously used an IUD, or had prior unsuccessful IUD.
De Nadai et al. [9]	Intracervical block for levonorgestrel-releasing intrauterine system placement among nulligravid women: A randomized double-blind controlled trial	Mar 2020	RCT	Brazil	University Hospital	Nulligravid women 18-45 years old who had never used any IUC	Women with medical conditions are considered category 3 or 4 for LNG-IUS according to WHO medical eligibility criteria. Additionally those with a history of chronic pelvic pain, any abnormalities or history of surgery of the cervix, illicit drug or alcohol use, known psychiatric disorders, chronic use of medications that could interfere with pain perception (eg, antidepressants and anticonvulsants), and current use of analgesics or anti-inflammatory agents.
Envall et al. [10]	Intrauterine mepivacaine instillation for pain relief during intrauterine device insertion in nulliparous women: a double-blind, randomized, controlled trial.	Feb 2019	RCT	Sweden	-	Nulliparous women 18 years or older desiring any type of IUD for contraception.	Previous ionization, known cervical stenosis, signs of ongoing genital infection, known uterine abnormality, bleeding disorder, or any local anesthetic contraindication.
Abd Allah et al. [11]	Dual-responsive lidocaine in situ gel reduces pain of intrauterine device insertion	Jan 2018	RCT	Egypt	Assiut Womens Health Hospital	Menstruating women, non-pregnant, aged 18-49 years old did not receive any analgesics or misoprostol in the 24 hours prior to insertion.	Uterine abnormalities such as fibroids, adenomyosis, endometrial lesions, and a history of lidocaine allergy.
Rapkin et al. [12]	Self-administered lidocaine gel for intrauterine device insertion in nulliparous women: a randomized controlled trial	Sep 2016	RCT	United States of America	-	English-speaking nulliparous women aged 14–50 years requesting insertion of either of the two IUDs available in the United States.	Known allergy or hypersensitivity to lidocaine or other amino amide local anesthetics, pregnancy in the previous 6 weeks, prior IUD use or failed IUD insertion, use of narcotic or benzodiazepine medication within the previous 24 hours.
Yaron et al. [13]	Safety and efficacy of a suction cervical stabilizer for intrauterine contraceptive device insertion: Results from a randomized, controlled study	Mar 2023	RCT	Switzer-land	Hospital	Women 18 years old or older.	Contraindications for IUD insertion, currently on oral anticoagulants, history of cervical operations or vaginal bleeding of unknown origin, or receiving analegsics within 12 hours of the procedure.
Micks et al. [14]	The effect of nitroglycerin on the IUD insertion experience in nulliparous women: a pilot study	Mar 2014	RCT	United States	OHSU and Planned Parenthood Columbia Willamette Clinic	18-45 year old females requesting IUD	Previous pregnancy beyond 20 weeks, previous IUD placement or attempted IUD placement, previous cervical cold knife cone or loop electrosurgical excision procedure, contraindication to LNG-IUS or nitroglycerin, history of hypertension or hypotensive disorder, history of migraine, cluster headaches or vascular headaches, and blood pressure less than 90/55 or greater than 150/100 in office prior to speculum exam.
Hylton et al. [15]	Cold Compress for Intrauterine Device Insertional Pain: A Randomized Control Trial.	Jun 2020	RCT	United States of America	Clinic	nonpregnant women, 18 years or older, placement of IUD for contraception	Decision impaired, imprisoned, non-English speaking, or under the age of 18 years.
Benazzous et al. [16]	Effects of virtual reality on pain during intrauterine device insertion: a randomized controlled trial	Nov 2023	RCT	France	Family planning center	All women over the age of 18	<18 yo, under legal guardianship, pre-existing dizziness, severe facial wounds, hx of epilepsy **use of pain medication was NOT part of exclusion criteria**
Samy, et al. [17]	Benefits of Self-administered Vaginal Dinoprostone 12 Hours before Levonorgestrel-releasing Intrauterine Device Insertion in Nulliparous Adolescents and Young Women: A Randomized Controlled Trial.	Aug 2020	RCT	Egypt	tertiary referral hospital	Nulliparous women, aged 18-22 years, with no current pregnancy or pregnancy within the past 6 weeks.	Diagnosed chronic pelvic pain (endometriosis, dysmenorrhea, IBS, interstitial cystitis), had taken analgesics within 24 hours of enrollment, had a history of previous IUD insertion, uterine or cervical anomaly or fibroids, unexplained vaginal bleeding, pelvic inflammatory disease or cervical infection, known allergy or contraindications to dinoprostone or IUD placement.
Samy et al. [18]	Prophylactic vaginal dinoprostone administration six hours prior to copper-T380A intrauterine device insertion in nulliparous women: A randomized controlled trial.	Mar 2020	RCT	Egypt	hospital	Nulliparous women requesting copper T 380A intrauterine contraception were offered to share in the trial if they were 18 years of age or older, had a negative pregnancy test, and had no previous pregnancies beyond 13 6/7 weeks of gestation.	We excluded women with the following conditions: pelvic inflammatory disease diagnosed within the last three months, active vaginitis or cervicitis, currently pregnant or pregnant within six weeks of study entry, had a history of cervical surgery. Also, we excluded women with undiagnosed abnormal uterine bleeding, World Health Organization Medical Eligibility Criteria category 3 or 4 precautions to a copper IUD, had a previous attempted or successful IUD insertion, fibroids or other uterine abnormalities distorting uterine cavity, a known allergy or contraindication to dinoprostone.
Ashour et al. [19]	Comparative efficacy of vaginal misoprostol vs vaginal dinoprostone administered 3 hours prior to copper T380A intrauterine device insertion in nulliparous women: a randomized controlled trial	Apr 2020	RCT	Egypt	tertiary referral hospital	Nulliparous women aged 18-25 years, requesting Copper-T380A IUD insertion as a contraceptive who had a negative pregnancy test result.	women with the following criteria; parous women, currently pregnant or have had a pregnancy within the past 14 days, untreated active cervicitis or vaginitis, pelvic inflammatory disease in the last 3 months, analgesic or anxiolytic use within 24 hours before the procedure, undiagnosed abnormal uterine bleeding, history of a cervical procedure such as cone biopsy, loop electrosurgical excision procedure, or cryotherapy, a known uterine anomaly or fibroid distorting uterine cavity, allergy or contraindications to dinoprostone or misoprostol such as glaucoma, asthma, uncontrolled diabetes and hypertension, hepatic and renal impairments, and contraindication to IUD use as defined in categories 3 and 4 of the updated medical eligibility criteria for contraceptive use

Quality Assessment of Studies

The quality of the 16 eligible studies was evaluated using the quality assessment feature on Covidence. Each of the studies was assigned a quality assessment value out of the possible poor, fair, and good ratings and assessed based on randomization, treatment of concealment, blinding of participants and personnel, blinding of outcome assessors, incomplete outcome data, selective outcome reporting, and other extra sources of bias. Each of the studies utilized in this review received a quality assessment value of good.

Level of Pain Control with Oral Analgesics

The use of oral analgesics, specifically 800 mg of ibuprofen one hour prior to the procedure, is currently recommended prior to an IUD insertion. However, the data surrounding the clinical significance of pain reduction with the use of oral analgesics are contradictory, with some studies supporting the use of oral analgesics and other studies showing no significant pain reduction with the use of oral analgesics. The use of 50 mg of tramadol one hour prior to IUD insertion was not found to have any clinical significance on pain reduction during the IUD insertion procedure (p=0.610) [4]. However, it was found that 550 mg of naproxen sodium taken prior to the insertion procedure led to a clinically significant reduction in pain post IUD insertion with average visual analog scale (VAS) scores of 26 and 16.5 for the control and treatment groups, respectively (p=0.01). However, this intervention did not provide any significant pain relief during cervical grasping (p=0.97) or during the actual insertion procedure (p=0.89) [5].

Level of Pain Control with Cervical Blocks

Cervical blocks are also a method of pain control that can be offered to individuals undergoing an IUD insertion. However, the use of a cervical block is not a standard procedure as a method of pain control. Studies that measured pain management with cervical blocks show inconsistent results. One study found that using 20 cc of buffered 1% lidocaine injected into the cervix led to a clinically significant reduction in pain during IUD insertion with average VAS scores for the control and treatment groups being 54 and 33, respectively (p=0.001). This study also showed a statistically significant reduction in pain post IUD insertion with the average VAS scores for the control and treatment groups being 27 and 12, respectively (p=0.005). However, there was no significant reduction in pain with cervical grasping when 1% lidocaine was injected into the cervix (p=0.268) [6]. Another study found that the use of 2% lidocaine injected into the cervix did not lead to any significant reduction in pain during IUD insertion (p=0.4) or post IUD insertion (p=0.1) [7]. Alternatively, another study found that the use of 3.6 mL of 2% lidocaine was associated with a significant reduction in pain with cervical grasping, IUD insertion, and pain control post IUD insertion. This study compared three groups: a group receiving a sham block, a group receiving a lidocaine block, and a group receiving no intervention. This study reported pain levels based on participants' classification of pain as mild, moderate, or severe pain. During tenaculum placement, 2% of the participants who received 2% lidocaine reported severe pain, while 30.2% of participants in the sham block group reported severe pain, and 15.2% of participants who received no intervention reported severe pain (p<0.0001). During IUD insertion, 26.5% of participants receiving the 2% lidocaine experienced severe pain, 59.4% of participants receiving the sham block experienced severe pain, and 50.5% of the participants who received no intervention experienced severe pain (p<0.0001) [8]. Another study compared the use of sole usage of 800 mg of ibuprofen with the usage of a combination of 800 mg of ibuprofen with 1 mL of 1% lidocaine at the tenaculum site and 4.5 mL of 1% lidocaine at the cervicovaginal junction. The results from this study show that the average VAS scores during tenaculum placement were 60 and 23.5 for the control and treatment groups, respectively (p<0.001). The average VAS scores during IUD insertion were 71.5 and 30 for the control and treatment groups, respectively (p<0.001). The average VAS scores post IUD insertion were 29 and 5 for the control and treatment groups, respectively (p<0.001) [9]. Lastly, a study comparing the use of 10 mL of 1% mepivacaine to placebo did not result in any significant pain management during cervical grasping (p=0.487), IUD insertion (p=0.062), or post IUD insertion (p=0.545) [10]. Furthermore, the injection of lidocaine, or another analgesic, into the cervix causes pain when the needle penetrates the cervix. For this reason, the use of lidocaine in the form of gel was also studied in order to prevent the pain that is associated with injecting analgesics into the cervix. The first study analyzing the use of analgesic gel found that using dual responsive in situ lidocaine gel led to a significant reduction in pain with cervical grasping, IUD insertion, and post IUD insertion. The average VAS scores for the control and treatment groups with cervical grasping were four and two, respectively (p=0.001). The average VAS scores for the control and treatment groups with IUD insertion were six and three, respectively (p=0.001). The average VAS scores for the control and treatment groups post IUD insertion were 2.5 and 1, respectively (p=0.001) [11]. The use of 4 mL of 2% lidocaine gel self-administered vaginally also led to a significant reduction in pain with cervical grasping, with average VAS scores of 56 and 32 for the control and treatment groups, respectively (p=0.03). However, there was no significant reduction in pain with IUD insertion (p=0.13) or pain post IUD insertion (p=0.52) [12].

Level of Pain Control with Innovative Techniques

There were also some studies that evaluated the level of pain control with innovative techniques that are not offered to individuals or routinely used during the IUD insertion procedure. One study examined the use of a cervical suction stabilizer rather than a tenaculum to grasp the cervix during IUD insertion. This study found that there was a clinically significant reduction in pain during cervical grasping with average VAS scores of 39 vs 11 for control and treatment groups (p<0.001), respectively, and during IUD insertion with average VAS scores of 57.8 vs 32.4 for control and treatment groups, respectively (p<0.001). However, there was no significant reduction in pain post IUD insertion when using a cervical suction stabilizer (p=0.100). In addition, this study found that there was significantly more cervical bruising with the use of the cervical suction stabilizer compared to the use of a tenaculum with 8 out of the 28 participants in the treatment group developing ecchymosis of the cervix, while none of the participants in the control group developed ecchymosis of the cervix [13]. Another study evaluated the use of nitroglycerin ointment on the cervix, which demonstrated no significant reduction in pain during the IUD insertion procedure (p=0.82) [14]. Another study evaluated the use of a cold compress applied to the abdomen for five minutes prior to IUD insertion. This study found no significant reduction in pain during the IUD insertion procedure (p=0.805) [15]. The last study compared the use of virtual reality and no standard treatment with just standard treatment. The findings from this study determined that there was significant pain reduction with the use of virtual reality during the IUD insertion procedure (p=0.54) [16].

Level of Pain Control with Cervical Softening and Dilation

Another method of pain control that is offered to some individuals is prostaglandins, which function to soften and dilate the cervix so that the insertion of the IUD is theoretically less painful. One research group completed three studies comparing the use of two prostaglandin E2 medications, dinoprostone, and misoprostol, administered at various time points prior to the insertion procedure. One of these studies compared vaginal administration of a placebo pill with vaginal administration of a 3 mg dinoprostone pill 12 hours prior to the IUD insertion procedure. The results from this study demonstrated a significant reduction in pain during cervical grasping and IUD insertion with the use of both dinoprostone. However, there was no significant reduction in pain post IUD insertion (p=0.59), and participants did experience significantly more abdominal cramping than the control group. During cervical grasping, this study found average VAS scores of 4.55 vs 2.97 for the control and treatment groups, respectively (p<0.001). During IUD insertion, this study found average VAS scores of 3.95 vs 2.83 for the control and treatment groups, respectively (p<0.01) [17]. The second study compared the use of 3 mg of dinoprostone administered vaginally six hours prior to the procedure with vaginal administration of a placebo pill and found that there was a significant reduction in pain with IUD insertion, with the average VAS scores with IUD insertion being 5 and 3.7, respectively (p<0.01). However, pain control with cervical grasping and post IUD insertion was not measured [18]. The third study compared the use of vaginally administered 3 mg of dinoprostone with the use of vaginally administered 3 mg of misoprostol with vaginally administered placebo, each administered three hours prior to the insertion procedure. This study found that there was a significant reduction in pain during cervical grasping, IUD insertion, and post IUD insertion. With cervical grasping, the average VAS scores for the control vs 3 mg misoprostol vs 3 mg dinoprostone were 4 vs 3.2 vs 2.2, respectively (p<0.001). With IUD insertion, the average VAS scores were 4.4, 2.4, and 3.1, respectively (p=0.02 ). Post IUD insertion, the average VAS scores were 3.4, 2.7, and 2.0, respectively (p<0.001) [19].

Discussion

Current guidelines for pain management during intrauterine device insertions only indicate the administration of 800 mg of ibuprofen one hour prior to the procedure [2]. However, the research surrounding the advantages of using ibuprofen is contradictory, with not many patients having any significant pain reduction with the use of 800 mg of ibuprofen. In addition, other studies that measured the effects of oral analgesics did not find any clinically significant reductions in pain levels among participants, which suggests that oral analgesics are not a completely effective method to manage pain during IUD insertions [4,5]. The use of cervical blocks as a pain management technique is also contradictory. In one study, injection of 2% lidocaine into the cervix yielded clinically significant pain management during the IUD insertion procedure [8]. In addition, there was another study that used 800 mg of ibuprofen with a total of 4.5 mL of 1% lidocaine injected into the cervix, and this study showed that this combination significantly reduced pain during the IUD insertion procedure [9]. However, another study determining the effects of the injection of 2% lidocaine into the cervix showed that there was no clinically significant reduction in pain during any portion of the IUD insertion [7]. Due to the conflicting evidence, there is minimal support for the use of injected lidocaine into the cervix, especially given that the injection itself would cause the patient discomfort. However, one study determining the effects of dual responsive in situ lidocaine gel found that there was clinically significant pain reduction during the procedure. In addition, since this study used lidocaine gel on the participants rather than lidocaine injections, there is the additional benefit of no discomfort during the injection of lidocaine. For this reason, the application of lidocaine gel appears to be advantageous to an individual's pain management during the IUD procedure [11]. However, as there was only a singular study completed on the application of in situ lidocaine gel prior to IUD insertions, it would be beneficial to complete future research studies analyzing the effects of lidocaine gel during IUD insertion procedures in order to confirm the findings in the initial study.

New and innovative techniques being studied for the first time were also included in this review. One of those new techniques was the use of a cervical suction stabilizer, rather than a tenaculum, to grasp the cervix. According to patients, one of the most painful steps of the IUD insertion procedure is when the tenaculum is used to grasp the cervix. This study used suction, rather than a tenaculum, to grasp the cervix in an attempt to mitigate the pain associated with the use of the tenaculum. The results from this study showed a clinically significant decrease in pain during both cervical grasping and IUD insertion. However, it is to be noted that there were more instances of cervical ecchymosis with the use of the cervical suction stabilizer rather than the tenaculum [13]. Despite the resulting ecchymosis, the benefits of pain management with the cervical suction stabilizer make this technique a good option for providers looking to improve patient satisfaction during IUD insertion. Other innovative techniques such as using virtual reality, using cold compresses on the abdomen, and using nitroglycerin ointment on the cervix were also studied [14-16]. However, none of these studies demonstrated any clinically significant changes in pain during the IUD insertion procedure. Therefore, these techniques do not have any evidence supporting their use in the clinical setting. However, because these are innovative techniques with the potential for improvement, other studies could be done to measure the effects of these techniques as they improve. Specifically, virtual reality is an up-and-coming technology that is continually making advances. As these advances continue, there may be more benefits of using virtual reality as both a relaxation and pain management technique for IUD insertions in the future.

Lastly, the use of prostaglandins showed advantageous effects on pain management during IUD insertion. Both the use of vaginal dinoprostone and vaginal misoprostol showed clinically significant reductions in pain during the IUD insertion procedure. There were three different studies comparing these medications to a placebo, and they were tested when given at three hours, six hours, or 12 hours prior to the procedure. All three studies showed clinically significant improvement in pain during the procedure, suggesting that there is no advantage or disadvantage to administering these medications at different time intervals [17-19]. In addition, this is one of the options that actually provides benefits, in regards to pain management during the IUD insertion procedure, and should therefore be heavily considered as an option for pain control. However, these medications cause additional discomfort in the way of abdominal cramping. Thus, if these medications were to be utilized as a method of pain management during IUD insertion procedures, the benefits would have to be weighed with the disadvantages of the added abdominal cramping. In addition, the three studies comparing the use of dinoprostone and misoprostol to placebo were only completed by one research group. For this reason, bias needs to be considered as a possibility for the positive outcomes of the studies [17-19]. However, because there were positive outcomes to the studies, it would be beneficial to complete future studies analyzing the use of dinoprostone and misoprostol during IUD insertions to accurately determine whether or not the use of these medications would actually be of clinical benefit to patients receiving an IUD insertion.

Although there were some studies that found significant results in pain management during IUD insertions, further studies should be done to strengthen the power of these results since many of the studies that were included in this review contain low sample sizes. Abd Ellah et al., who found a significant reduction in pain during the IUD insertion procedure, had a sample size of 48 [11]. Samy et al., who found a significant reduction in pain during IUD insertions when using dinoprostone or misoprostol, had sample sizes of 118, 200, and 130 in three studies, respectively [17-19]. Yaron et al., who found a significant reduction in pain during IUD insertions when using a cervical suction grasper, had a sample size of 55 [13]. In order to support the findings in these studies, further critical evaluation of the use of these techniques during IUD insertions is necessary.

Limitations

The inclusion criteria indicated that articles had to be peer-reviewed, written in English, and written within the last 10 years. Based on the inclusion criteria, the scoping review was limited to analyzing only 16 articles. The limited amount of articles consequently led to a limited sample size and outcomes assessed for each modality of pain management. Limitations specific to the various articles are outlined in the results section above. Additionally, the study criteria limited the participants to be women of at least 18 years of age and nulliparous.

Future Research Implications

Future research may benefit by including a measurement of preconceived pain or anxiety about IUD insertion to assess whether they play a role in the overall pain experienced during IUD insertion. Researchers could also combine oral analgesic pain control and either cervical block or cervical softening to optimize the management of pain control during and after IUD insertion. This approach has the potential to improve patient satisfaction with IUD insertion. Additionally, in the future, there have been some studies that have found that transcutaneous electrical nerve stimulation (TENS) may play a beneficial role in decreasing pain during IUD insertion [20]. Overall, a decrease in pain and an increase in patient satisfaction may ultimately encourage more women to get an IUD, which can play a beneficial role in women who experience menstrual complications such as cramping and anemia.

## Conclusions

Pain associated with IUD insertion remains a persistent concern for women. However, there are studies that demonstrate a significant reduction of pain with both medications and innovative techniques. For the innovative techniques, using a cervical suction grasper is effective at reducing pain during cervical grasping and reducing pain during the IUD insertion. For the medications, lidocaine gel appears to be excellent at reducing pain during all aspects of the IUD insertion procedure and has minimal side effects. Lidocaine injections in combination with 800 mg of ibuprofen also have been shown to provide significant pain relief during the IUD insertion, but there is additional pain during the lidocaine injection that individuals would not experience when using lidocaine gel. Lastly, prostaglandins, specifically misoprostol and dinoprostone, have been shown to also significantly reduce the pain associated with the IUD insertion procedure, although these drugs also cause a side effect of abdominal cramping. Overall, there are many viable options for pain control for IUD insertions. However, further study on these various techniques should be completed in order to further support a standardized recommendation for pain control for individuals who are receiving an IUD.
